# Evaluating a Bilingual, Community-Based Skin Cancer Education Program in an Underserved Hispanic Population: An Item Response Theory Analysis

**DOI:** 10.7759/cureus.115970

**Published:** 2026-09-08

**Authors:** Smaran Marupudi, Natasha N Quailes, Jared Hensley, Juan C Lopez-Alvarenga, Maria Villegas

**Affiliations:** 1 Dermatology, The University of Texas Rio Grande Valley School of Medicine, Edinburg, USA; 2 Transitional Year Residency, South Texas Health System Graduate Medical Education (GME) Consortium, Edinburg, USA; 3 Population Health and Biostatistics, The University of Texas Rio Grande Valley School of Medicine, Edinburg, USA; 4 Dermatology Surgical Specialty and Musculoskeletal Integrated Specialty Unit (MSK ISU), The University of Texas Rio Grande Valley School of Medicine, Harlingen, USA

**Keywords:** educational intervention, health disparities, hispanics, irt, rio grande valley, skin cancer prevention

## Abstract

Background: Skin cancer prevention is often underemphasized in medically underserved regions such as the Rio Grande Valley (RGV), where residents, predominantly Hispanic and lower-income individuals, face limited access to dermatologic care and may be unaware of their risk for skin cancer.

Methods: The intervention included a bilingual educational session with a board-certified dermatologist and a seven-item knowledge survey administered before and immediately after the session, adapted from a previously validated instrument. A convenience sample of 73 participants completed both surveys. The instrument, adapted for this population, was evaluated using a two-parameter Item Response Theory (IRT) model to estimate participants’ underlying knowledge proficiency (theta) beyond raw scores. Panel regression was used to assess how demographic variables, including age, gender, education, income, rural residence, and language, predicted knowledge change. A post hoc content validity index (CVI) was conducted with 10 experts from dermatology and public health.

Results: Results revealed small-to-moderate improvements in knowledge scores across the sample, with question 5 (tan damage) and question 6 (sunscreen application) exhibiting the highest information at lower knowledge levels. Bilingual language use and higher education levels were positively associated with higher knowledge scores, while older age and rural residence were linked to smaller improvements. Variation between theta values and raw scores highlighted variability in learning outcomes, supporting the use of IRT to capture nuanced change. Results of the CVI revealed scores of 0.99 for relevance, 0.90 for clarity, 0.96 for representativeness, and 0.96 for cultural appropriateness.

Conclusion: This intervention demonstrates the feasibility of a bilingual, community-driven education effort and utilized IRT to identify strengths and weaknesses of individual survey items. It provides evidence to guide refinement of future skin cancer educational assessment tools and interventions for underserved Hispanic populations such as the RGV.

## Introduction

The Lower Rio Grande Valley (LRGV), comprising Hidalgo, Cameron, Starr, and Willacy counties, is a medically underserved, majority-Hispanic region along the US-Mexico border with high poverty and low insurance coverage [1,2]. This demographic is particularly significant in dermatology, as some individuals in Hispanic communities believe that their natural pigmentation protects them from skin cancer [3,4]. Furthermore, studies have shown Latinos are likely to be less cognizant of the causes and associated symptoms of skin cancer [3], have lower perceived risk, and have less engagement in preventative behaviors [4]. These population-level risk factors, combined with elevated ultraviolet (UV) indices in southern Texas (11.5: “Extreme Category”) [5,6], contribute to delayed diagnoses and poorer outcomes compared to non-Latino counterparts. Studies indicate that individuals in the LRGV tend to be diagnosed with melanoma at older ages [7], experience higher melanoma-related mortality rates [8], and have shorter survival times following a diagnosis of non-melanoma skin cancer compared to populations in other regions of Texas [9].

To address gaps in skin cancer prevention in this setting, we implemented a bilingual skin cancer education and screening intervention during Operation Border Health Preparedness (OBHP)-a week-long public health initiative that offers free walk-in care to underserved communities in the LRGV. The primary objective of this prospective, single-arm pre-post feasibility pilot study was to evaluate implementation of a bilingual community-based skin cancer educational intervention and assess short-term change in skin cancer knowledge using an Item Response Theory (IRT)-derived theta score. Secondary analyses evaluated changes in raw knowledge scores and individual item performance. Exploratory analyses examined associations between demographic characteristics and knowledge outcomes, including whether selected demographic characteristics modified pre-post change.

## Materials and methods

Study design

Ethics and Study Sample

The University of Texas Rio Grande Valley Institutional Review Board (IRB) approved the study (IRB-24-0122). We conducted a prospective, single-arm pre-post feasibility pilot study during OBHP in McAllen, Texas, from July 23 to 25, 2024.

After informed consent was obtained, the participants completed a pre-survey assessing knowledge, attitudes, and practices related to skin cancer and sun safety. The knowledge section was modeled on the Skin Cancer and Sun Knowledge Scale, and questions were adapted to a sixth-grade reading level or below in order to align with the anticipated low-health literacy level [10]. The Spanish version of both surveys was reviewed by a Spanish language expert prior to implementation.

The primary outcome was the change in knowledge from immediately before to immediately after the intervention, measured using the IRT-derived theta score. Secondary outcomes included changes in raw knowledge scores and individual questionnaire-item performance. Exploratory analyses examined whether participant demographic characteristics were associated with knowledge outcomes and whether selected characteristics modified pre-post change. The study inclusion criteria were being 18 years or older, being a registered patient at Operation Border Health, being of either sex, and signing the informed consent.

Procedure

Participants received an immediate response-dependent, automated explanation after answering each item (Appendix). Specifically, after each response, participants were shown an explanation corresponding to whether their answer was correct or incorrect. If participants had questions about an item or its explanation, study staff provided verbal clarification limited to the information and concepts contained within the corresponding automated explanation. Following the pre-survey, participants underwent a skin consultation with a board-certified dermatologist who reinforced skin cancer risk factors, identification of suspicious lesions, and sun-protection strategies.

After the consultation, participants immediately completed the post-survey, where they also received automated feedback. Because these components occurred sequentially within a single community-based encounter, pre-post knowledge change was interpreted as two components delivered during the same encounter: (1) automated, response-dependent feedback with verbal clarification limited to the content of the provided explanations and (2) a consultation with a dermatologist reinforcing skin cancer risk factors.

All study materials, including the informed consent documents, were provided in both English and Spanish. Spanish-speaking participants were assisted throughout the study by staff who were native Spanish speakers, ensuring full comprehension. Patients were compensated for their participation.

Content validity index

Post Hoc Validity

We also conducted an IRB-exempt post hoc content validity assessment based on the original items. A panel of 10 experts in dermatology or public health was recruited based on their subject-matter expertise in dermatology or public health. Panelists completed an online survey and were asked to rate each item for relevance, clarity, representativeness, and cultural appropriateness using a 4-point Likert scale (1 = not relevant/needs major revision to 4 = excellent item with no revision needed).

Panel members ranged in age from 34 to 69 years. The highest completed degrees included Master’s (n = 2), Doctorate (n = 2), and Professional (n = 6). Seven experts were female, and three were male, with years of experience ranging from three to 35. Since surveys were bilingual, language background was also assessed. Six participants reported speaking primarily English at home, one primarily spoke Spanish, and three reported speaking both English and Spanish. Item-level content validity indices (I-CVI) were calculated as the proportion of experts rating each item as either 3 (adequate) or 4 (excellent) on a 4-point Likert scale, and the scale-level content validity index (S-CVI/Ave) was computed as the average of the I-CVI values across all items.

Statistical analysis

We included all eligible participants who attended the intervention event during the data collection period. Descriptive statistics were used for demographic variables using mean (standard deviation (SD)) for continuous variables or frequency (percentages) for nominal variables. Effect sizes for paired comparisons were calculated using Cohen’s dz (mean differences divided by SD of differences).

We calculated the McNemar odds ratio (OR) (95% confidence interval (CI)) of attaining the correct answer in the post-test, which reflects the odds of improvement from pre- to post-test on a per-question basis. This statistical procedure is commonly used to evaluate changes in outcomes within matched-pairs data, such as repeated measures on the same participants. We utilized a two-parameter IRT model to estimate participants’ underlying skin cancer knowledge from their response patterns, represented by the parameter (θ). Because of the limited pilot sample size, the two-parameter logistic (2PL) analysis was considered exploratory. As a sensitivity analysis, a more parsimonious one-parameter logistic (1PL) model was fitted to the same seven baseline items and complete-case sample and compared with the 2PL model using likelihood-ratio testing and Akaike and Bayesian information criteria (AIC and BIC). Item parameter uncertainty was evaluated using 95% CIs.

Unlike traditional percentage correct scoring, IRT accounts for differences in question difficulty and each question’s ability to distinguish between participants with higher and lower knowledge (discrimination) [10,11]. To evaluate the performance of individual questionnaire items, we examined item characteristic curves, which show the probability of answering a question correctly across different θ values. We also examined item information curves, which indicate the knowledge levels at which each question is most useful for accurately estimating participants’ knowledge. For instance, items that peak near θ = 0 are most informative for participants with average knowledge levels, while items peaking at negative or positive values help assess lower or higher knowledge levels, respectively.

Pre- and post-intervention item responses were reshaped into long format and jointly calibrated using an exploratory 2PL IRT model, rather than fitting separate models at each assessment. Joint calibration placed the pre- and post-intervention theta estimates on a common latent metric. Post-test responses to Q2 were excluded from IRT estimation because of a potentially problematic item, while baseline Q2 responses were retained. Participant-specific theta estimates were then obtained for each assessment and analyzed longitudinally using a random-effects linear panel regression estimated by maximum likelihood. Participant ID was specified as the panel variable and assessment occasion (pre vs. post) as the time variable, allowing the participant-specific random effect to account for within-participant dependence in the longitudinal analysis. Demographic characteristics were included as fixed covariates (gender, age, education, household income, rural residence, and primary language spoken at home) to obtain an adjusted estimate of pre-post change. Given the pilot sample size, the joint IRT calibration did not explicitly model within-participant dependence at the item-response level, and formal longitudinal measurement invariance was not established; therefore, longitudinal comparisons of IRT-derived theta were considered exploratory. The fully adjusted longitudinal theta model included 69 participants, each contributing pre- and post-intervention observations (138 observations total). Observations with missing values for the outcome or any covariate included in a given model were excluded from that model; no imputation was performed.

A leave-one-covariate-out sensitivity analysis was performed using the same complete-case analytical sample (n = 69) to assess the stability of the adjusted pre-post effect across alternative model specifications [12]. Education, household income, rural residence, insurance, and language were individually excluded from the fully adjusted model. Model fit was summarized using AIC and BIC, and the pre-post coefficient and 95% CI were compared across specifications. We present the p-values and BIC for sensitivity analysis of the models (Table 1).

**Table 1:** Factors associated with the ability to respond correctly after an educational intervention on skin cancer prevention Two models are presented: score coefficient displays raw score changes from the questionnaire, and theta coefficient represents theta estimate changes from pre-test to post-test. *p < 0.05 was considered significant.

Variables	Score coefficient	p-value*	Theta coefficient	p-value*
Age	-1.4 (-4.4, 1.6)	0.363	-0.006 (-0.022, 0.01)	0.445
Sex	Reference: male			
Female	-1.2 (-10, 7.6)	0.792	-0.013 (-0.06, 0.034)	0.59
Education	Reference: elementary			
High school (HS)	12.5 (2.6, 22.5)	0.013	0.055 (0.001, 0.108)	0.044
Graduate HS/more	14.7 (0.9, 28.6)	0.037	0.06 (-0.014, 0.134)	0.115
College/more	10.4 (-0.3, 21)	0.056	0.048 (-0.009, 0.105)	0.098
Household income	Reference: <$20,000			
$20,000 to $34,999	-5.3 (-14.5, 3.9)	0.255	-0.016 (-0.066, 0.033)	0.513
$35,000 to $49,999	-3.3 (-17.5, 10.9)	0.649	-0.006 (-0.082, 0.07)	0.875
$50,000 to $100,000	-3 (-16, 10)	0.647	-0.005 (-0.074, 0.065)	0.898
>$100,000	-21.9 (-51.1, 7.3)	0.141	-0.087 (-0.243, 0.069)	0.275
Living in a rural area	Reference: urban			
Rural	-10.2 (-18.6, -1.8)	0.018	-0.034 (-0.079, 0.011)	0.136
Insurance	Reference: private			
Public insurance	-5.5 (-16.7, 5.7)	0.337	-0.021 (-0.081, 0.039)	0.492
No insurance	-6.8 (-16.2, 2.7)	0.159	-0.021 (-0.071, 0.03)	0.418
Language in home	Reference: only Spanish			
Spanish >> English	-9.9 (-21.4, 1.6)	0.09	-0.078 (-0.14, -0.017)	0.012
Spanish == English	13 (2.8, 23.2)	0.013	0.068 (0.013, 0.122)	0.015
Spanish << English	7.1 (-4.4, 18.6)	0.226	0.043 (-0.019, 0.104)	0.175
English only	5.1 (-10, 20.3)	0.506	0.03 (-0.051, 0.11)	0.472
Test #1	Reference: test #1			
Test #2	10.3 (5.5, 15.2)	<0.001	0.077 (0.054, 0.101)	<0.001
Constant	66.2 (49.1, 83.4)	<0.001	0.779 (0.687, 0.87)	<0.001

Exploratory effect-modification analysis

To determine whether pre-post changes in knowledge differed across demographic groups, additional random-effects panel models included interaction terms between assessment time (pre vs. post) and each demographic characteristic. These models retained the remaining demographic variables as covariates. The interaction terms were interpreted as differences in pre-post change between each category and its reference group, rather than as overall between-group differences in theta. Adjusted marginal predictions were estimated from the interaction models to visualize both predicted theta at each assessment and the adjusted pre-post change within demographic categories. Given the pilot sample size and small numbers within some categories, these interaction analyses were considered exploratory (see Figures 1, 2). All analyses were performed with Stata version 18.5 (StataCorp College Station, TX, USA). A formal sample size calculation was not conducted, as this is a feasibility pilot study.

**Figure 1:**
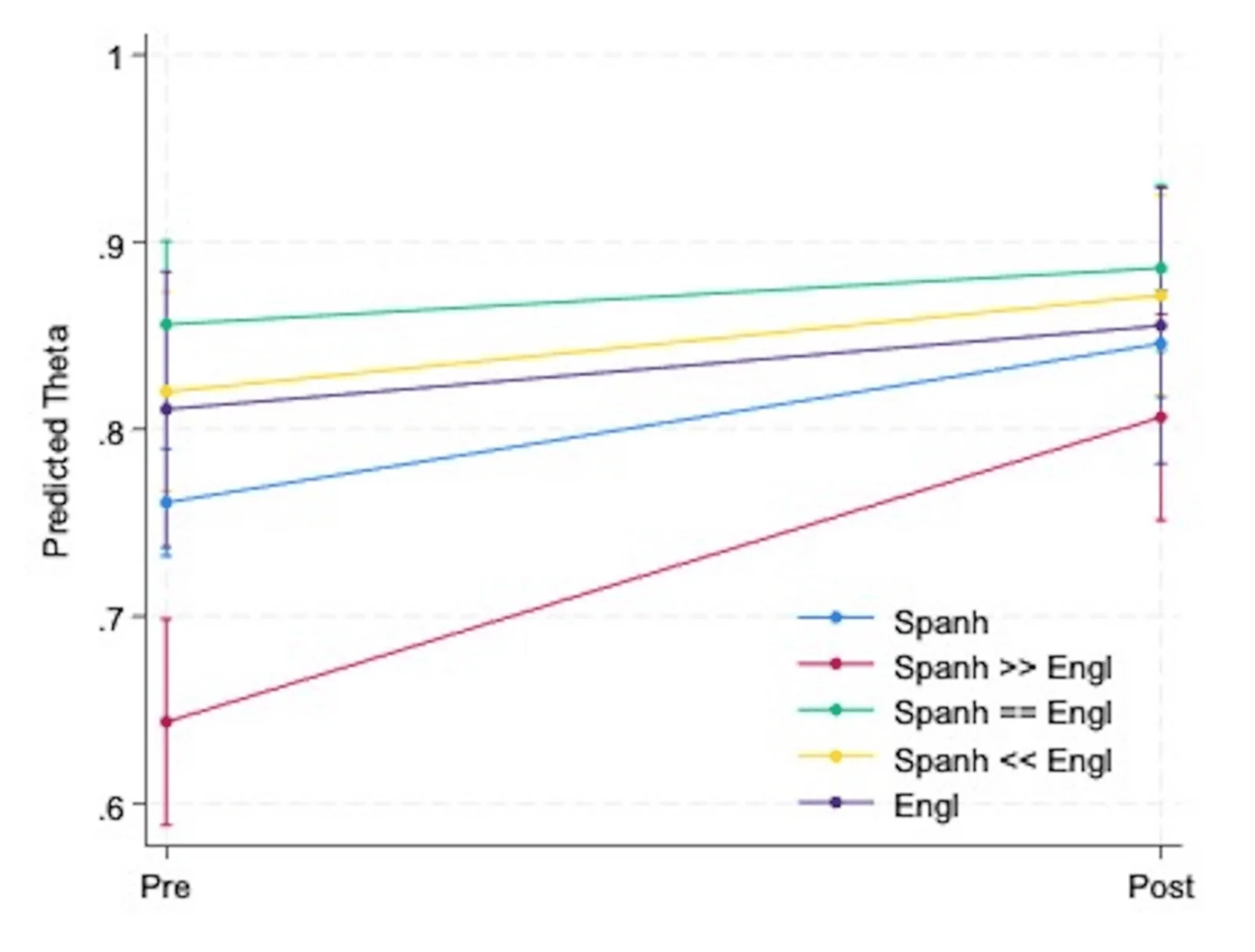
Adjusted pre- and post-intervention theta estimates according to language spoken at home Predicted theta values (95% CI) were obtained from the random-effects panel regression including the interaction between assessment time and home-language category, adjusted for age, sex, education, household income, rural residence, and insurance status. CI: confidence interval

**Figure 2:**
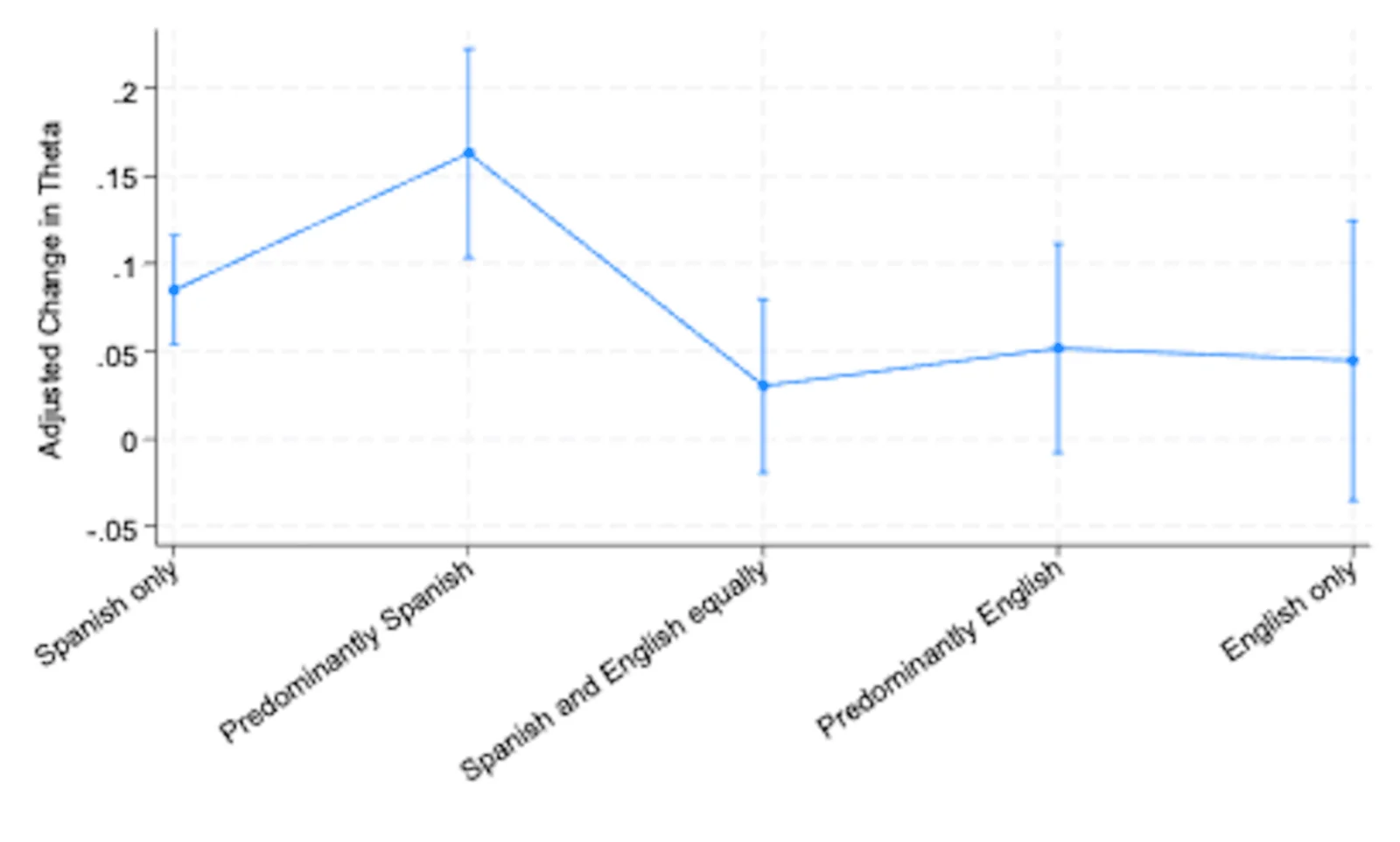
Adjusted pre-post change in theta according to language spoken at home Points represent the model-estimated change in theta from pre- to post-intervention within each home-language category, with error bars (95% CI). Estimates were derived from the random-effects panel model including the time-by-language interaction and adjusted for age, sex, education, household income, rural residence, and insurance status. Positive values indicate improvement in theta after the educational exposure. CI: confidence interval

## Results

Of the 73 participants (mean age 40.7 years, SD 12.7), 57 (78%) were women, 33 (45%) reported education beyond high school, 18 (25%) lived in rural areas, and 26 (36%) were unemployed. Spanish was the primary language spoken at home by 34 (47%) participants. Among the 70 participants with evaluable questionnaire data, the mean six-item knowledge score, excluding Q2 because of a potentially problematic item, increased from 62.9% (SD 21.1) at baseline to 80.7% (SD 19.4) post-intervention, with a paired mean increase of 17.8 percentage points (95% CI 12.2-23.5; p < 0.001) with Cohen’s d of 0.76. In a sensitivity analysis retaining Q2, the seven-item score increased from 63.5% (SD 21.4) to 74.7% (SD 17.1), with a paired mean increase of 11.2 percentage points (95% CI 6.1-16.4; p < 0.001) with Cohen’s d of 0.52.

In the baseline sensitivity analysis (n = 70), the 2PL model provided a better likelihood-based fit than the 1PL model (LR χ²(6) = 23.36, p = 0.0007) and had a lower AIC (510.05 vs. 521.41). BIC slightly favored the more parsimonious 1PL model (539.40 vs. 541.53). Taken together, these findings suggested heterogeneity in item discrimination but also supported cautious interpretation given the small sample and additional complexity of the 2PL model.

The item information curves for the dichotomic and multiple responses (Figure 3) demonstrate the measurement precision of each item across knowledge levels (θ), with higher information values indicating greater precision in a question's ability to estimate participants’ knowledge at a given ability level. As seen in Figure 3a, questions 1 (sunburn) and 3 (dark skin) demonstrated lower information values across the theta spectrum, indicating limited precision. While question 2 (sun exposure) demonstrated moderate precision at lower knowledge values, it was excluded from the post-test IRT analysis due to inconsistent responses-limiting its interpretability. As seen in Figures 3a, 3b, question 5 (tan damage) and question 6 (sunscreen application) exhibited the highest information at lower knowledge levels (theta ≈ -1). Questions 4 and 7 provided relatively modest information across a broad range of theta values.

**Figure 3:**
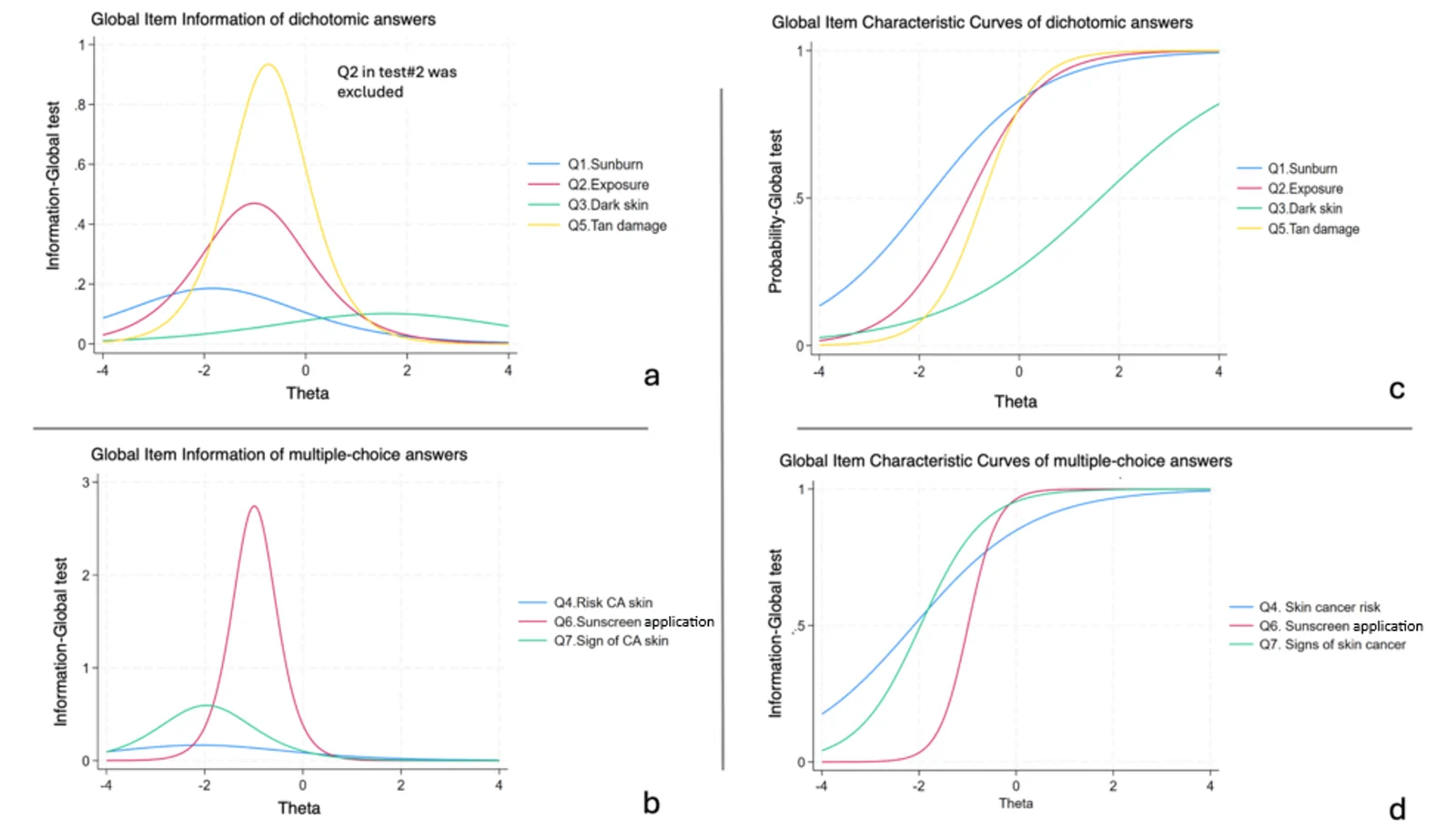
Information and characteristic curves for four true-or-false questions and three multiple-choice questions assessing skin cancer knowledge across participant ability levels (theta) This figure displays information curves for four true-or-false questions (Q1: sunburn, Q2: exposure, Q3: dark skin, and Q5: tan damage) and three multiple-choice questions (Q4: risk of skin cancer (CA skin), Q6: sunscreen application, and Q7: signs of skin cancer (CA skin)) across participant ability levels (theta).

As seen in Figure 3c, participants with low theta values already have a high probability of answering Q1 correctly. Questions 2 (only pre-test) and 5 indicate moderate difficulty, with steep slopes indicating good discrimination. Question 3 had the greatest difficulty, but weaker discrimination, as exhibited by the right-shifted curve and gradual slope. In Figure 3d, question 4 was relatively easy, with participants of lower θ having a higher probability of answering correctly. Question 7 demonstrated higher difficulty and stronger discrimination than question 4. Question 6 exhibits the steepest item characteristic curve, indicating the strongest discrimination (Figure 3).

The McNemar test for paired samples showed that most participants improved in correct responses in the second test. However, question 4 did not show a significant change in accuracy (OR = 0.88, p = 1.0), consistent with its low difficulty parameter (-2.1, representing a very easy item) and acceptable discrimination (0.82) (Table 2).

**Table 2:** Item-level accuracy, matched odds ratios, and exploratory IRT parameters before and after the educational session Values are presented as the percentage of correct responses at pre- and post-test (pre/post). Pre-post changes were evaluated using the McNemar test with matched odds ratios and 95% CI. Discrimination and difficulty are exploratory two-parameter logistic IRT estimates (95% CI). Higher discrimination indicates greater ability to distinguish participants across levels of the latent knowledge trait, while difficulty represents the item's location on the theta scale. *p < 0.05 IRT: Item Response Theory; CI: confidence interval

Questions	Correct % (pre-test/post-test)	Odds ratio (95% CI)	Discrimination (95% CI)	Difficulty (95% CI)
Q1. If you are not usually exposed to the sun, being severely sunburned two or three times during your life will probably increase your chances of skin cancer. (T/F)	73/87	4.3 (1.2, 23.7)*	0.86 (0.18, 1.54)	-1.84 (-3.1, -0.6)
Q2. The only way a person can get skin cancer is from too much exposure to the sun. (T/F)	67/39	0.2 (0.06, 0.53)*	1.37 (0.2, 2.5)	-1.0 (-1.6, -0.4)
Q3. People with dark skin cannot get skin cancer. (T/F)	11/44	4.8 (2.0, 14.2)*	0.64 (-0.003, 1.3)	1.6 (0.1, 3.2)
Q4. Which of the following increases your risk of skin cancer? Having had three severe sunburns in your past; having a family history of skin cancer; both of these options; neither of these options	83/81	0.88 (0.27, 2.8)	0.82 (0.1, 1.5)	-2.1 (-3.6, -0.6)
Q5. A tan is a sign that the skin is damaged. (T/F)	55/86	6.3 (2.2, 24.7)*	1.9 (0.6, 3.3)	-0.7 (-1.1, -0.4)
Q6. When should sunscreen be applied for best protection? Just before you go in the sun; 15-30 minutes before going in the sun; within 15-30 minutes after going in the sun	70/91	16 (2.5, 671)*	3.3 (-0.1, 6.7)	-1 (-1.3, -0.6)
Q7. Which of the following could be a sign of skin cancer? A sudden or gradual change in a mole's appearance; a sore that does not heal; both of these options; neither of these options	85/95	4.5 (0.93, 42.8)	1.5 (0.3, 2.7)	-1.9 (-2.9, -1.0)

Panel regression results indicated a substantial increase in theta (knowledge scores) on the post-test. Participants with a high school education had significantly higher theta estimates (β = 0.055, p = 0.044) from pre- to post-test compared to those with an elementary education. Bilingual fluency was also associated with greater theta estimates (β = 0.068, p = 0.015) compared to only Spanish (β = -0.078, p = 0.012), despite participating in the same intervention (Table 1). Living in a rural area was associated with significantly lower post-test raw scores (β = -10.2, p = 0.018), though its negative association with theta knowledge estimates did not reach statistical significance (β = -0.034, p = 0.136). Other non-significant predictors were age, sex, household income, and insurance status. Time-by-demographic interaction analyses were conducted separately to evaluate whether the magnitude of pre- to post-test change differed across demographic groups (Figures 1, 2, 4).

**Figure 4:**
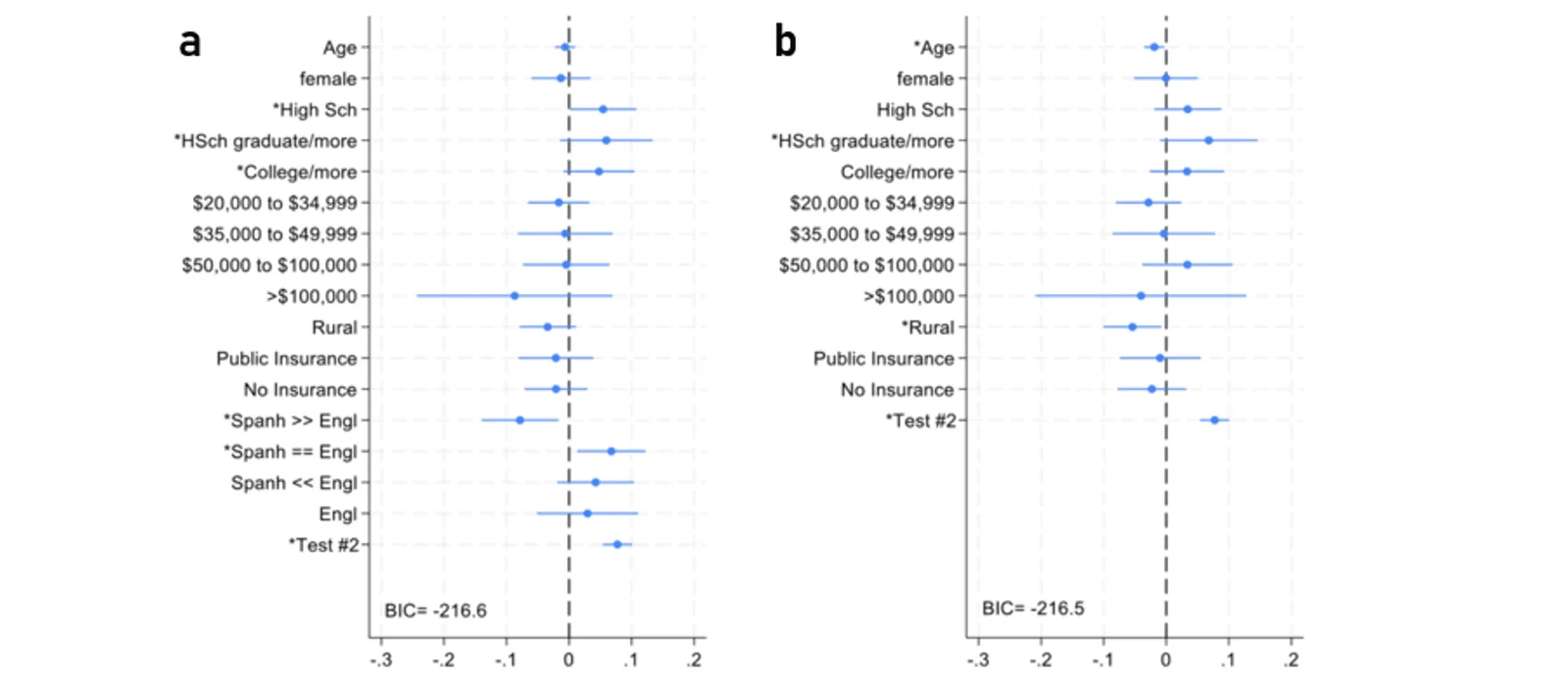
Coefficients adjusted with a panel (longitudinal analysis) The model in (a) includes language spoken at home and shows the strongest association for participants who spoke Spanish and English equally (p < 0.05). In the sensitivity analysis (b), language was excluded, revealing significant effects for age and rural residence (p < 0.05). Both models had comparable Bayesian information criterion (BIC) values (-216.6 vs. -216.5), indicating a similar overall model fit. Asterisks (*) indicate statistically significant coefficients for theta (95% confidence interval (CI) does not include zero). Spanish >> English: predominantly Spanish-speaking; Spanish == English: bilingual (Spanish and English equally); Spanish << English: predominantly English-speaking; Engl: only English-speaking

Table 3 shows that the adjusted pre-post increase in theta was highly robust to alternative covariate specifications. Across sensitivity models, AIC ranged from −281.2 to −263.4 and BIC from −234.3 to −216.5. The model excluding household income had the lowest AIC and BIC, although exclusion of individual covariates did not alter the estimated pre-post effect.

**Table 3:** Sensitivity analysis of the adjusted pre-post change in IRT-derived theta across alternative covariate specifications The sample size for this analysis was constant at n = 69 with data in all variables. Observe that the pre-post theta effect was stable when covariates were removed. It was remarkably stable. The beta coefficient (95% CI) is shown. AIC: Akaike information criterion; BIC: Bayesian information criterion; CI: confidence interval; IRT: Item Response Theory

Sensitivity model n = 69	AIC	BIC	Test (post vs. pre) β	Key change from full model
Full model	-275.2	-216.6	0.077 (0.054, 0.101)	Reference
Excluding education	-275.4	-225.6	0.077 (0.054, 0.101)	Pre-post estimate unchanged
Excluding income	-281.2	-234.3	0.077 (0.054, 0.101)	Pre-post estimate unchanged; lowest BIC
Excluding rural residence	-274.3	-218.6	0.077 (0.054, 0.101)	Pre-post estimate unchanged
Excluding insurance	-278.2	-225.5	0.077 (0.054, 0.101)	Pre-post estimate unchanged
Excluding language	-263.4	-216.5	0.077 (0.054, 0.101)	Pre-post estimate unchanged

Content validity assessment

The scale demonstrated excellent content validity. The S-CVI/Ave values were 0.99 for relevance, 0.90 for clarity, 0.96 for representativeness, and 0.96 for cultural appropriateness. All items exceeded the acceptable I-CVI threshold of 0.78. Minor wording revisions were made to two items based on expert feedback.

## Discussion

Key findings

The successful implementation of a targeted educational intervention within an existing community screening program suggests that concise educational assessments can be feasibly integrated to address knowledge gaps in skin cancer prevention in a medically underserved, predominantly Hispanic region. Specifically, the overall improvement in mean knowledge scores and increase in scores among bilingual participants and individuals with higher education indicates that targeted educational sessions can be an effective first step toward improving knowledge and may potentially facilitate sun-exposure behaviors in high-risk groups [13]. Immediate knowledge acquisition is a necessary precursor to informed preventive behavior, but whether the observed gains translate into sustained knowledge or behavioral change requires longitudinal evaluation. In addition, the observed improvement in knowledge should be interpreted in the context of the complete educational encounter, which included immediate survey-based feedback followed by dermatologist-led education. These components were intentionally integrated within the one-day community intervention, and their individual effects cannot be separated. Nevertheless, the findings demonstrate that participants could acquire and demonstrate skin cancer knowledge during a brief educational encounter in this community setting. These results highlight the value of culturally tailored interventions in improving health literacy in underserved communities, particularly those at higher risk for skin cancer.

IRT analysis provides actionable guidance for question refinement. The information and characteristic curves both indicate that Q5 and Q6 were the strongest-performing components in the questionnaire, providing the most information for participants with lower baseline knowledge and effectively distinguishing participants. These characteristics make these questions particularly valuable for educational assessments in underserved Hispanic populations, where baseline skin cancer knowledge is often limited. However, questions 1, 3, 4, and 7 provided limited information and poorer discrimination, although for various reasons. Some items appeared too easy to effectively distinguish among participants (ex., Q1 and Q4), whereas others may have assessed concepts that were either particularly challenging (Q3) or inadequately addressed during the intervention. These findings should be confirmed in a larger sample before finalizing questionnaire revisions. Importantly, qualitative patterns and overall aggregate data still reflected a net improvement in sun safety awareness and skin cancer risk comprehension. Overall, the IRT analysis demonstrated that the questionnaire was most sensitive for participants with lower baseline knowledge, consistent with prior reports of lower skin cancer knowledge in Hispanic populations.

Several factors influenced the effectiveness of the intervention. Participants fluent in both Spanish and English demonstrated the greatest improvement, demonstrating that bilingual communication may influence learning outcomes. Higher educational attainment was also associated with higher knowledge scores, emphasizing the importance of tailoring interventions to accommodate individuals with varying levels of formal education. Conversely, living in rural areas, being of older age, and having lower baseline education levels negatively impacted knowledge improvement. Of note, language influences knowledge gain in older residents and those in rural areas, highlighting the compound barriers that rural and older LRGV residents face.

Implications for practice and policy

These findings suggest that future dermatological interventions may benefit from culturally tailored teaching and assessment in minority-dense regions like the LRGV. The current literature has shown bilingual and simplified educational materials to be effective, and addressing barriers faced by rural populations through mobile clinics or community-based outreach programs could further reduce disparities [14-16]. These findings align with the Healthy People 2030 goal of increasing the proportion of adults who get recommended evidence-based preventive healthcare [17]. Furthermore, participants in our program showed measurable improvements in knowledge and awareness, aligning with prior evidence that bilingual and culturally tailored interventions-such as community health worker programs-are effective in reducing skin cancer disparities among Hispanic and migrant populations [18,19]. Given that Spanish-speaking patients have some of the lowest health literacy levels in the United States, and most dermatologic educational materials are written at inappropriate reading levels, our findings highlight a model for addressing both linguistic and literacy barriers [20,21]. Together, these findings support the integration of culturally tailored, educational interventions into existing community health outreach programs serving medically underserved populations.

Future directions 

Future actions will focus on validating and refining this questionnaire in larger populations to confirm the performance of the individual items identified through IRT analysis. Studies should determine if tailoring interventions to participant demographics improves learning outcomes compared to standard approaches. Longitudinal studies are needed to assess whether short-term knowledge gains reflect consistent sun-protective behaviors and earlier skin cancer detection. Finally, multi-center studies should evaluate whether integrating bilingual educational interventions into community-based screenings is feasible and effective across other medically underserved, Hispanic-predominant regions.

Barriers and challenges

While the study is strengthened by its use of advanced statistical tools like IRT and panel regression, limitations include a relatively small sample size to measure differences in all social conditions (e.g., rural area and economic status). Although the sample was enough to facilitate pilot pre- and post-assessment, it may have been underpowered for subgroup analyses. A technical limitation of this study was the potentially problematic item question 2 during post-test survey administration; however, the sensitivity analysis and conventional scores support post-intervention improvement. Another limitation was the lack of a control group, and the study may suffer from volunteer bias as the participant pool consisted of self-selected patients. In addition, we were unable to determine long-term behavior change. Future validation in a larger sample should evaluate item-parameter stability and measurement invariance across assessment occasions.

## Conclusions

We conclude that education-focused dermatology-related interventions along the US-Mexico border are an opportunity to address the risk of skin cancer among individuals living in the Rio Grande Valley. The survey instrument effectively detected changes in participant knowledge, and IRT identified revision opportunities for future assessment. The main factors affecting knowledge gains were primary language used at home, bilingual fluency, and attained education, with older age and rural residence representing secondary barriers. This study emphasizes the potential of culturally tailored education to bridge critical knowledge gaps regarding skin cancer risks in high-risk populations. Scaling such initiatives across the US-Mexico border region and beyond is essential to addressing preventable disparities in dermatological health outcomes.

## Figures and Tables

**Figure 5:**
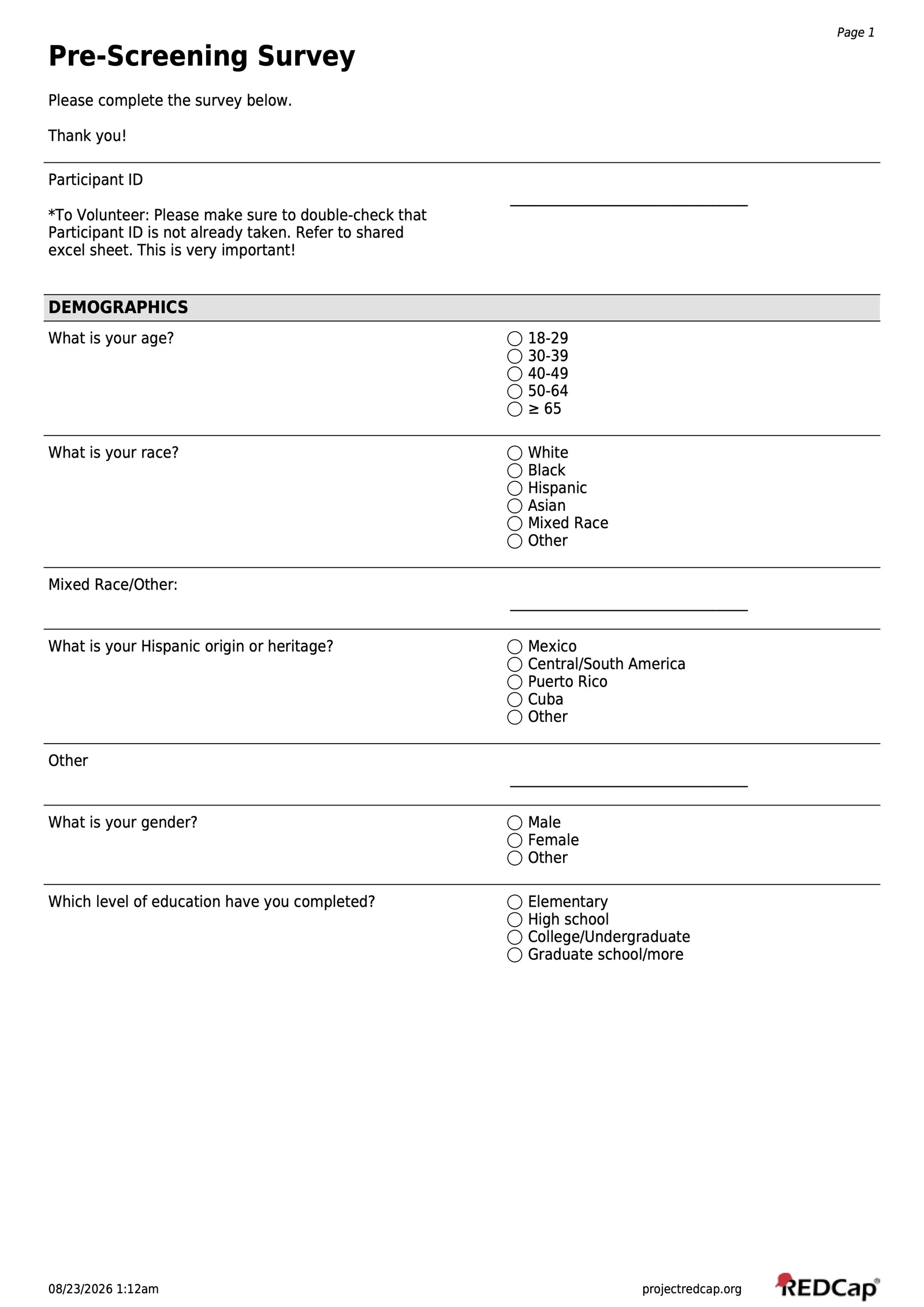
Pre-survey page 1

**Figure 6:**
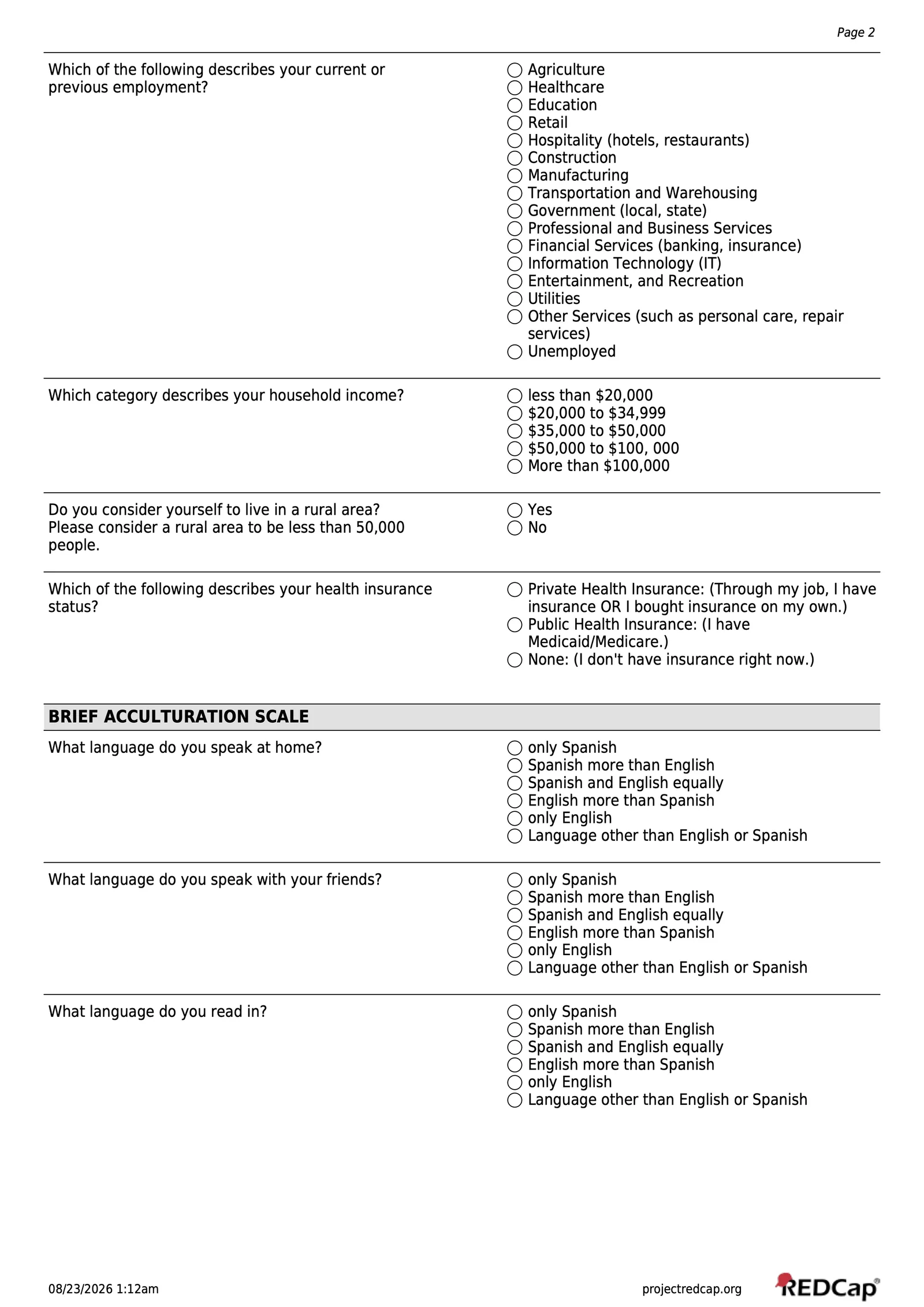
Pre-survey page 2

**Figure 7:**
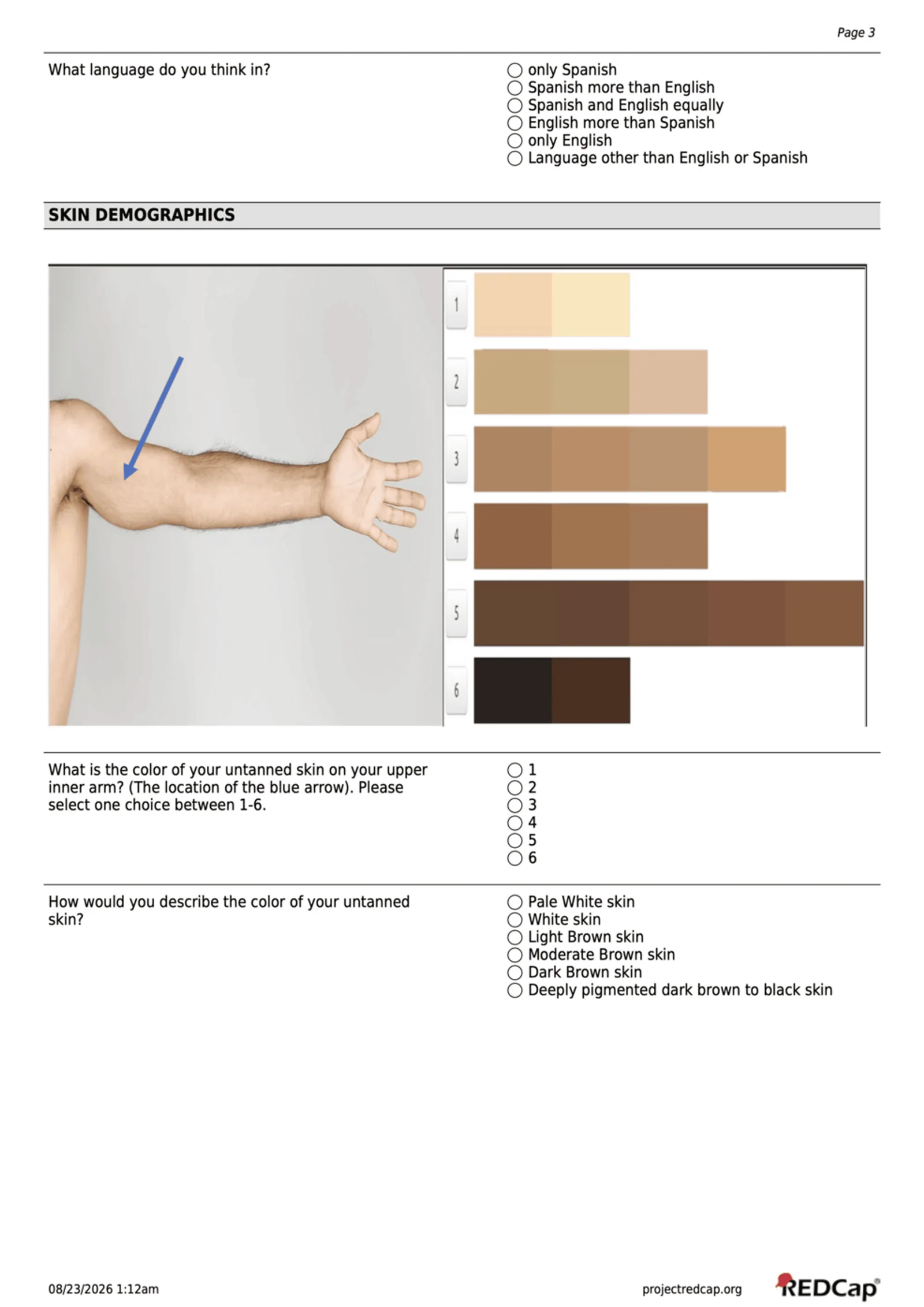
Pre-survey page 3

**Figure 8:**
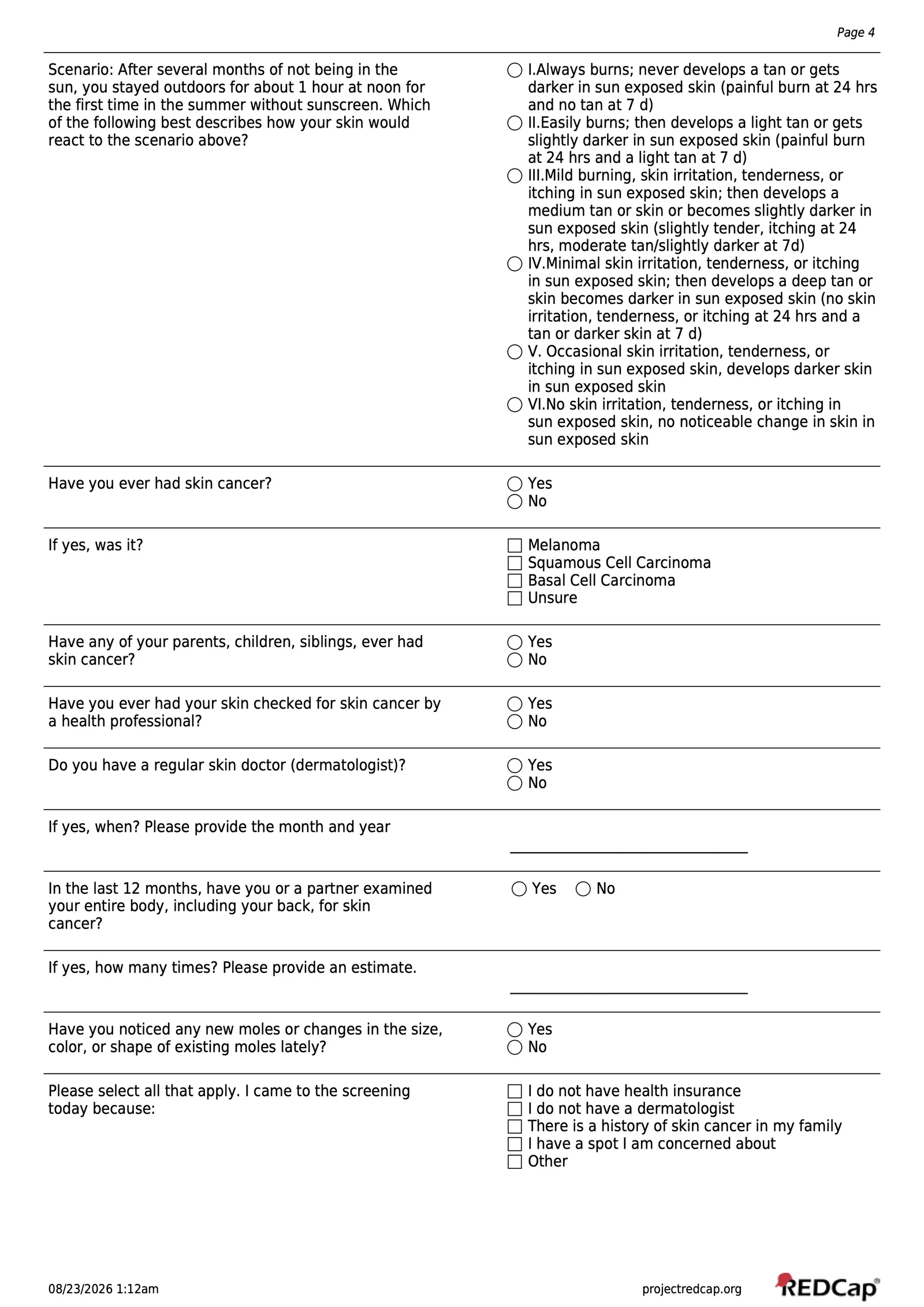
Pre-survey page 4

**Figure 9:**
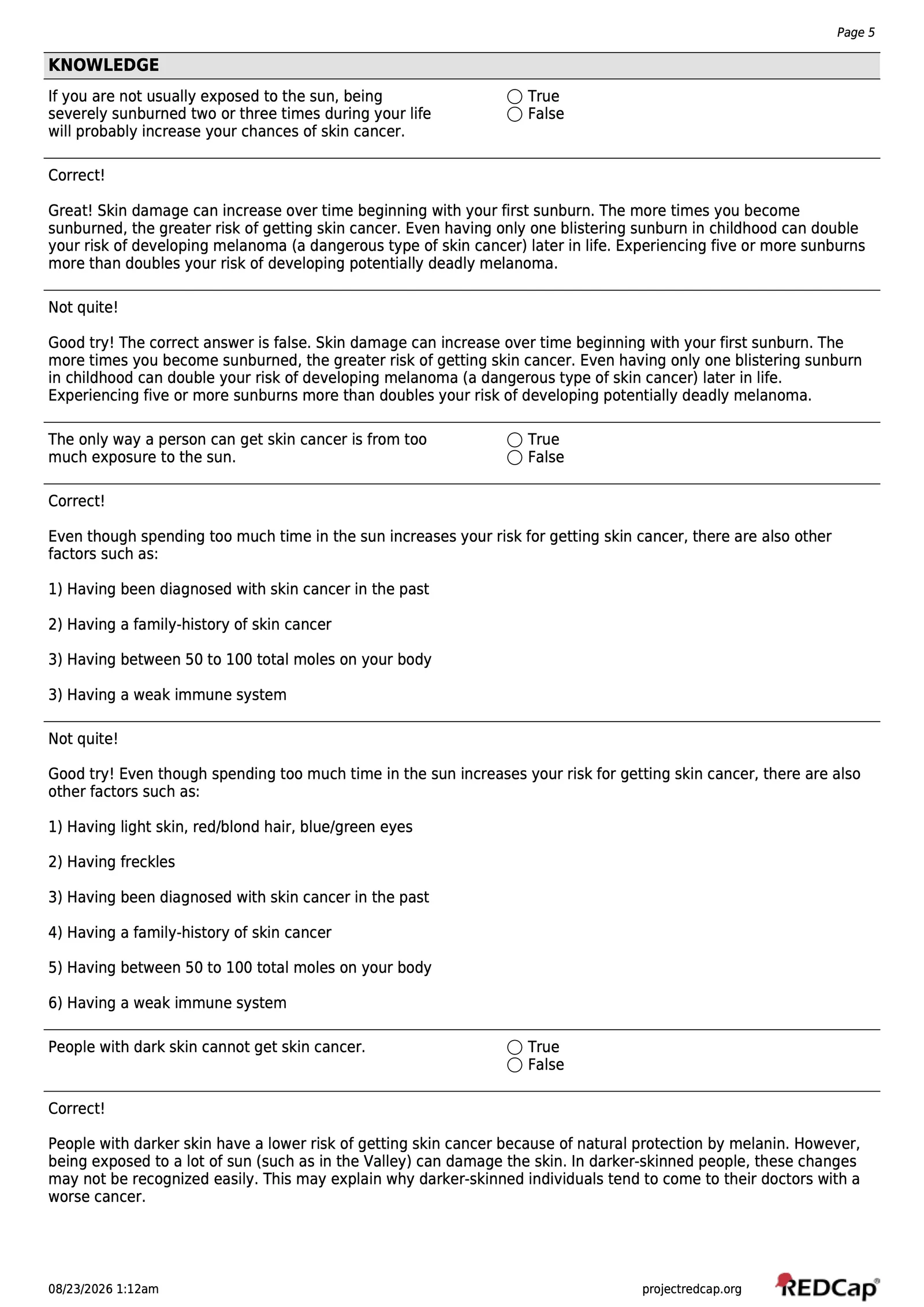
Pre-survey page 5

**Figure 10:**
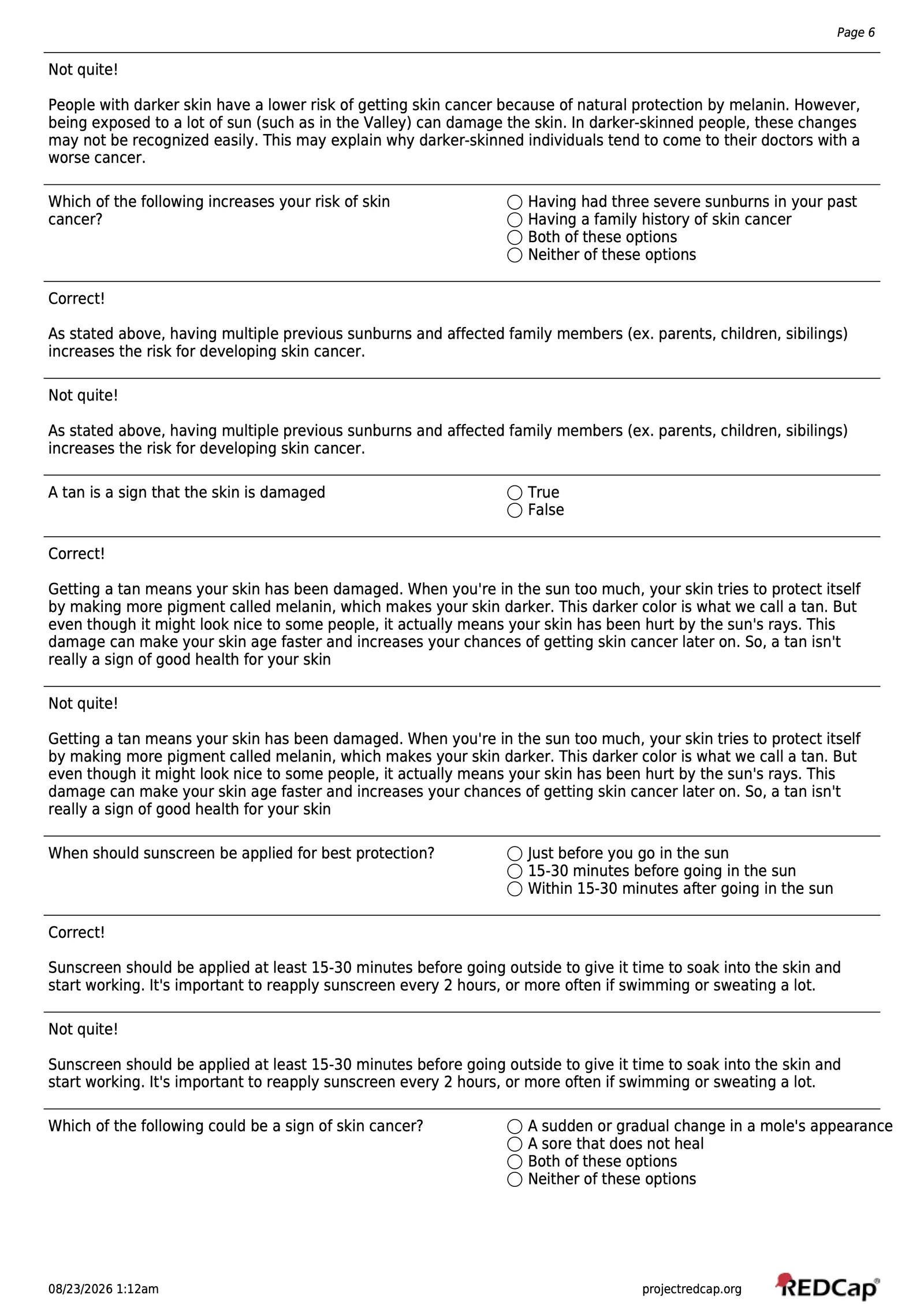
Pre-survey page 6

**Figure 11:**
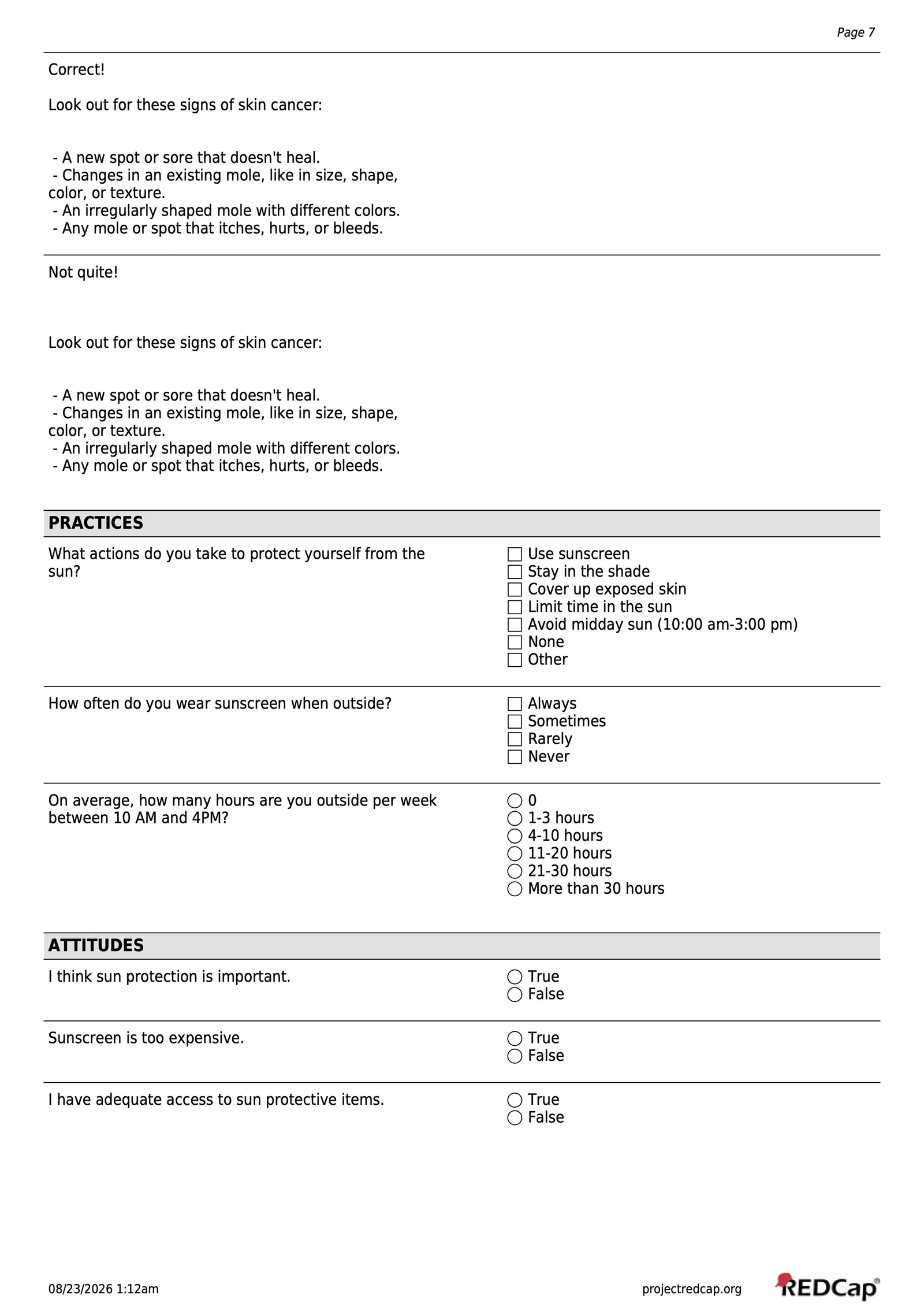
Pre-survey page 7

**Figure 12:**
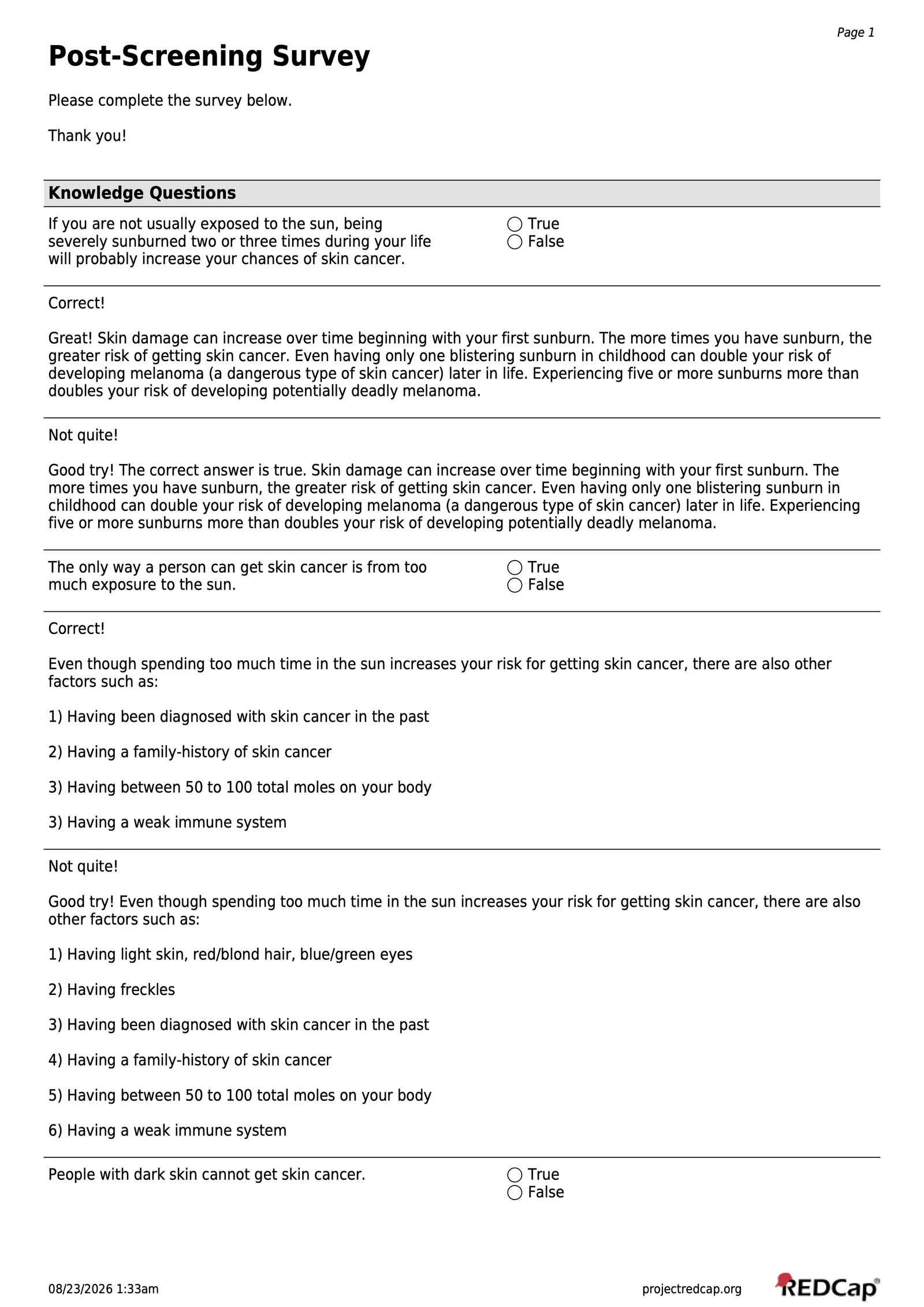
Post-survey page 1

**Figure 13:**
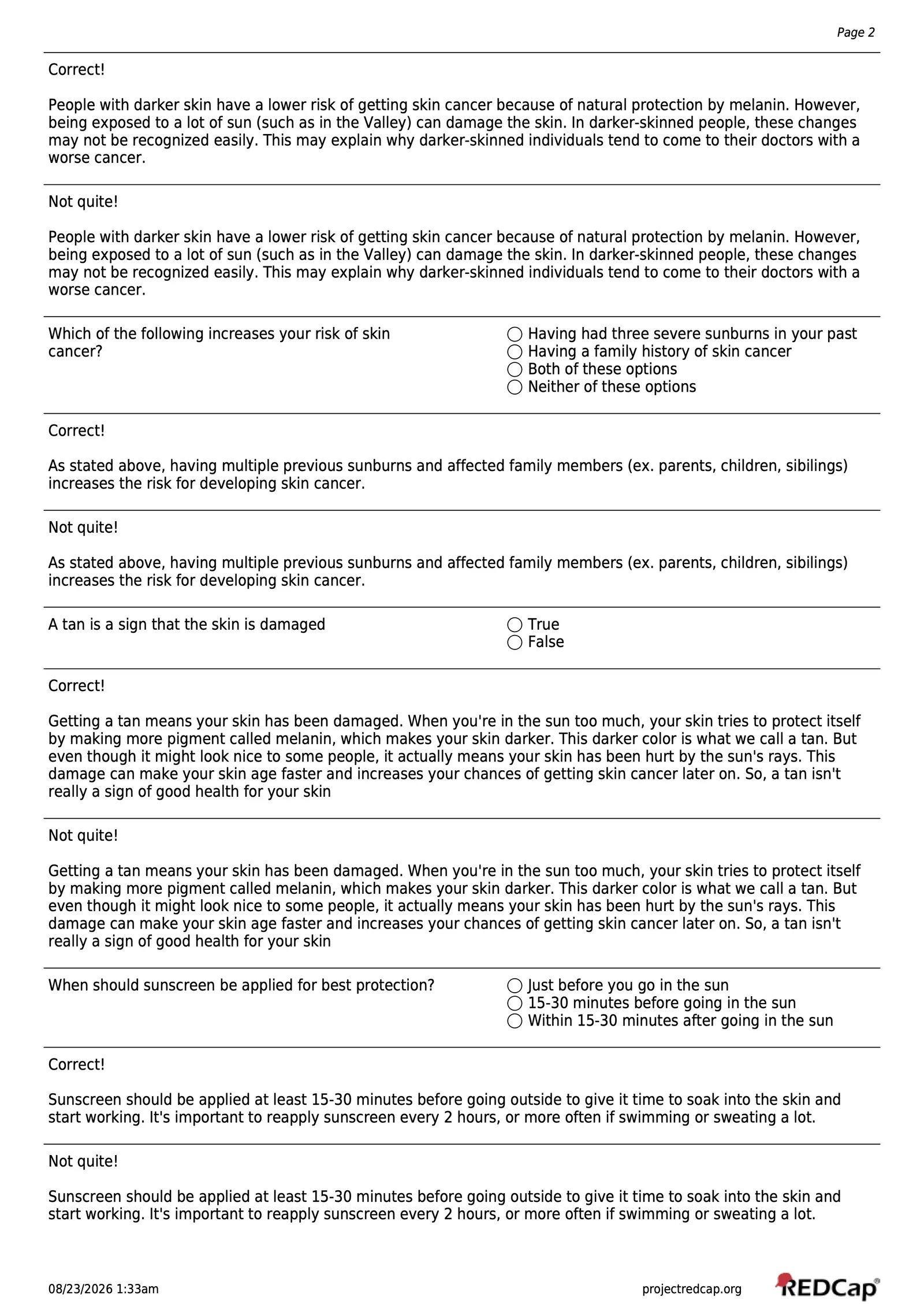
Post-survey page 2

**Figure 14:**
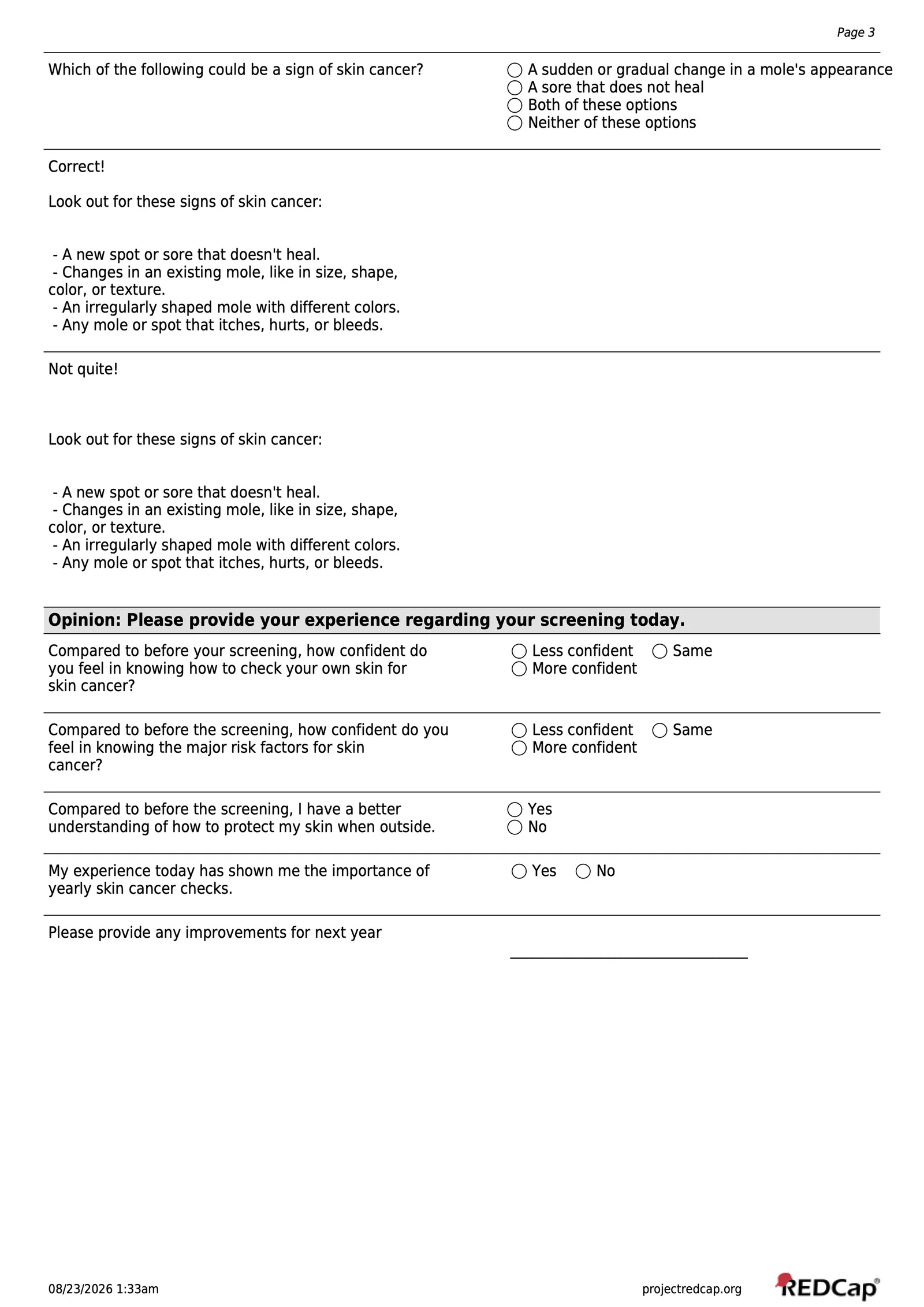
Post-survey page 3

## Appendices

Appendix A 

Appendix B 

Appendix C 

Appendix D

Appendix E 

Appendix F 

Appendix G 

Appendix H 

Appendix I 

Appendix J 
